# Developing potent PROTACs tools for selective degradation of HDAC6 protein

**DOI:** 10.1007/s13238-018-0602-z

**Published:** 2019-01-02

**Authors:** Zixuan An, Wenxing Lv, Shang Su, Wei Wu, Yu Rao

**Affiliations:** 1 MOE Key Laboratory of Protein Sciences, School of Life Sciences, Tsinghua University, Beijing, 100084 China; 2 MOE Key Laboratory of Protein Sciences, School of Pharmaceutical Sciences, MOE Key Laboratory of Bioorganic Phosphorus Chemistry & Chemical Biology, Beijing Advanced Innovation Center for Structural Biology, Tsinghua University, Beijing, 100084 China

**Dear Editor,**

Histone deacetylases (HDACs) are a family of enzymes that remove acetyl groups on histone and non-histone proteins, thereby playing a vital role in the modulation of gene expression and protein activity. Eighteen HDACs have been identified in human and subdivided into four classes including I, II (IIa, IIb), III and IV (Seto et al., 2014). Among them, HDAC6 is a unique IIb HDAC with dominant cytoplasmic localization and two functional catalytic domains. Besides the functions for deacetylation of histone, and modulation of α-tubulin, HSP90 and cortactin, HDAC6 also participates in protein trafficking and degradation, cell shape and migration (Valenzuela-Fernandez et al., 2008). The deregulation of HDAC6 is related to various diseases, such as neurodegenerative diseases, cancer and pathological autoimmune response (Batchu et al., 2016). Hence, it is especially important for directly controlling cellular HDAC6 protein levels to achieve therapeutic purposes. The traditional approaches of reducing cellular protein levels mainly rely on genetic modifications, such as RNA interference, transcription activator-like effector nucleases, recombination-based gene knockout and clustered regularly interspaced short palindromic repeats (CRISPR-Cas9) (Boettcher et al., 2015). However, these approaches have failed to a certain degree to achieve acute and reversible changes of gene function. Furthermore, the complications of potential genetic compensation and/or spontaneous mutations arising in gene-knockout models may lead to misinterpretations (Davisson et al., 2012; El-Brolosy et al., 2017). Therefore, it is urgent for developing a rapid, robust, and reversible approach to directly modulate HDAC6 protein levels.

Known as a chemical based protein knockdown strategy, PROteolysis-TArgeting Chimera (PROTAC) has emerged as a novel and powerful method for the degradation of interested proteins. The PROTACs are heterobifunctional molecules, which consist of three parts: a ligand for binding target protein, a ligand for recruiting E3 ligase and a linker connecting the two ligands (Lai et al., 2017). Consequently, the PROTACs mediated interaction of the target protein and a E3 ligase caused ubiquitination and subsequent degradation of the target protein by the ubiquitin-proteasome system (UPS) (Fig. 1A). It has been proved that PROTAC technology can achieve efficient degradation of proteins with excellent selectivity in a quick and direct manner (Yang et al., 2018; Zhou et al., 2018). Moreover, the PROTAC also worked well for mutated proteins (Sun et al., 2018). Herein, we report the development of HDAC6-targeting degraders based on the PROTAC strategy. The newly designed PROTACs induced significant degradation of HDAC6 in a panel of cell lines, exhibited excellent selectivity against other HDACs, and demonstrated efficient inhibition of cell proliferation. Besides, the degradation process was well illustrated by fluorescence-based visualization.

**Figure 1:**
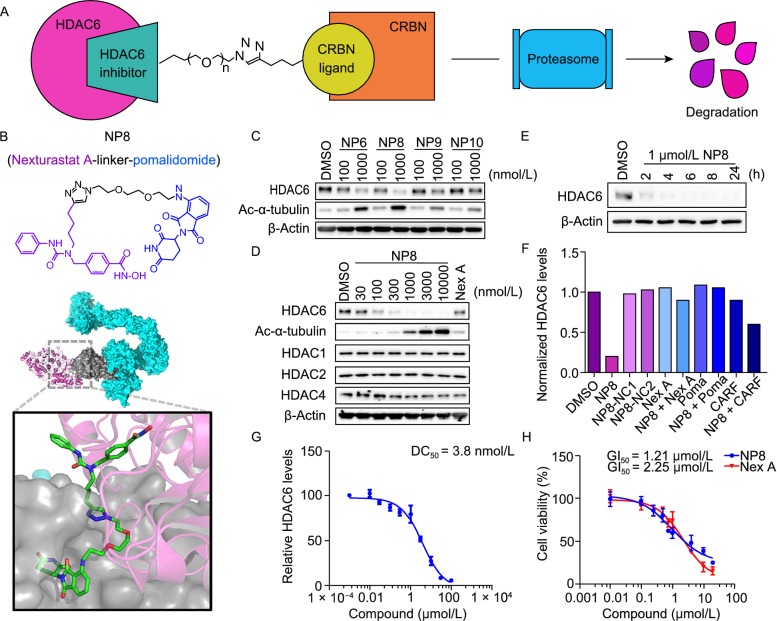
**Development of selective HDAC6-degrading PROTACs**. (A) The principle of PROTAC. (B) The structure of PROTAC, as shown in the upper portion. A binding mode of PROTAC (ball stick), HDAC6 (PDB 5G0J, purple) and CRL4-CRBN (PDB 2HYE and 4CI3, colored cyan and gray) was simulated by Pymol. (C) Screen for a potent HDAC6 degrader. HeLa cells were treated as indicated for 24 h. (D) Characterization of NP8-induced degradation in HeLa cells. The degradation of HDAC6 was in a dose-dependent manner. The HDAC1, HDAC2 and HDAC4 levels were not affected within 24 h. (E) NP8 caused fast degradation of HDAC6 within 2 h in HeLa cells. (F) NP8-NC1 and NP8-NC2 failed to degrade HDAC6. NP8 induced degradation was rescued by single introduction of Nexturastat A (Nex A, 300 nmol/L), Pomalidomide (Poma, 10 µmol/L) or Carfilzomib (CARF, 1 µmol/L). The concentration of NP8-NC1, NP8-NC2 and NP8 were 300 nmol/L. HeLa cells were treated with Carfilzomib for 6 h and 24 h for the rest. (G) The degradation of HDAC6 by titration of NP8 for 24 h in MM.1S cells. (H) MM.1S cells were treated with NP8 or Nexturastat A (Nex A) for 72 h. Cell viability was determined by CCK-8 assay (*n* = 3)

To design novel HDAC6-targeting PROTACs, we chose a selective HDAC6 inhibitor Nexturastat A (Nex A) as the HDAC6 binder (Bergman et al., 2012). According to the recently released co-crystal structure of HDAC6 in complex with Nex A (Miyake et al., 2016), the aliphatic chain was oriented outside of the ligand binding pocket. Based on the PROTACs design principles, Pomalidomide (Poma, a ligand for E3 ligase CRBN) was introduced onto the end of aliphatic chain of Nex A via different linkers (Lopez-Girona et al., 2012). As shown in the simulated diagram (Fig. 1B), the PROTACs should actively bind HDAC6 and CRBN simultaneously. The synthesis of the HDAC6 degraders was shown in Supplementary Materials (Scheme 1). Next, the resulting HDAC6-targeting PROTAC molecules were tested.

To evaluate the degradation capability of our PROTACs for HDAC6 protein, we analyzed the cellular levels of HDAC6 in HeLa cells by Western blot after incubation with four different PROTACs. It was found that all PROTACs can effectively induce HDAC6 degradation after 24 h. Among them, NP8 was the most potent degrader which can significantly reduce the HDAC6 protein level at 100 nmol/L (Fig. 1C). We then went on to evaluate the degradation potential of NP8 in a panel of cell lines from different origins. NP8 consistently induced significant degradation of HDAC6 in all the cell lines we tested, while the multiple myeloma cell line MM.1S exhibited the best sensitivity to NP8 (Fig. S1). The NP8-induced degradation was specific for HDAC6 since the other representative HDAC family members were not affected by NP8 treatment (Figs. 1D and S2). Time-lapse experiment showed that NP8 induced fast and effective degradation of HDAC6 in just 2 h post drug treatment (Fig. 1E). The half degradation concentration (DC_50_) of NP8 in MM.1S was 3.8 nmol/L (Fig. 1G). The proliferation of MM.1S was inhibited by NP8 in a dose-dependent manner, with significant and comparable level as the parental drug Nex A (Fig. 1H). To further characterize the working mechanisms for NP8, we applied two negative controls, NP8-NC1 and NP8-NC2. NP8-NC1 is an inactive analog of NP8 with a defect in binding CRBN, while NP8-NC2 is the simple conjugate of Poma and linker without Nex A (namely 3 in Scheme 1). Compared to NP8, these two control molecules both failed to induce HDAC6 degradation, implying the essence of simultaneous HDAC6 and CRBN binding for successful degradation (Fig. 1F). In parallel, NP8-induced HDAC6 depletion could be blocked by co-treatment of Nex A or Poma, again indicating NP8 needed binding of CRBN and HDAC6 to fulfil target degradation (Fig. 1F). Additionally, proteasome inhibitor Carfilzomib (CARF) could block the HDAC6 degradation, further demonstrating the dependence on proteasome for PROTAC (Fig. 1F) (Kuhn et al., 2007). Moreover, HDAC6 levels were quickly restored upon NP8 removal (Fig. S3). Collectively, these data demonstrated that the PROTAC NP8 represented a potent and specific degrader of HDAC6. Though small molecule inhibitors of HDAC6 have been studied extensively, there are still some problems remained to be solved, including the potential off-target effects, and the loss of efficacy to gene mutations (Batchu et al., 2016). Additionally, the non-enzymatic functions of HDAC6 cannot be affected by inhibitors. Compared with typical inhibitors, HDAC6-targeting degraders may have advantages over conventional inhibition by small molecules.

Next, to understand the dynamic details of PROTAC-induced HDAC6 degradation, direct visualization was applied for monitoring the process. With the potent and selective HDAC6-targeting degrader NP8, we were able to observe that how the HDAC6 degradation occurred under the treatment of NP8. We fused an enhanced green fluorescence protein (EGFP) to the N-terminal of HDAC6 to track the distribution and dynamics of HDAC6 in cells. The EGFP-HDAC6 showed an exclusively cytoplasmic distribution, consistent with reported HDAC6 subcellular localization (Kawaguchi et al., 2003) (Fig. 2A). When transfected into HeLa, the signals of EGFP-HDAC6 could be attenuated upon the treatment of NP8 (Fig. 2B) and the fusion proteins were indeed degraded under NP8 induction (Fig. 2C). When the signals from several single cells were monitored in time-lapse manner, the significant reduction of mean fluorescence intensities could be observed in a consecutive time window (Fig. 2D). To be noted, the response of EGFP-HDAC6 to NP8 was somehow not as rapid as endogenous HDAC6, possibly due to much higher expression level after transfection. Nonetheless, these data suggested that EGFP-HDAC6 was a responsive indicator for PROTAC-induced degradation and further visualization approaches may also be realized via combination of NP8 and other methods.

**Figure 2:**
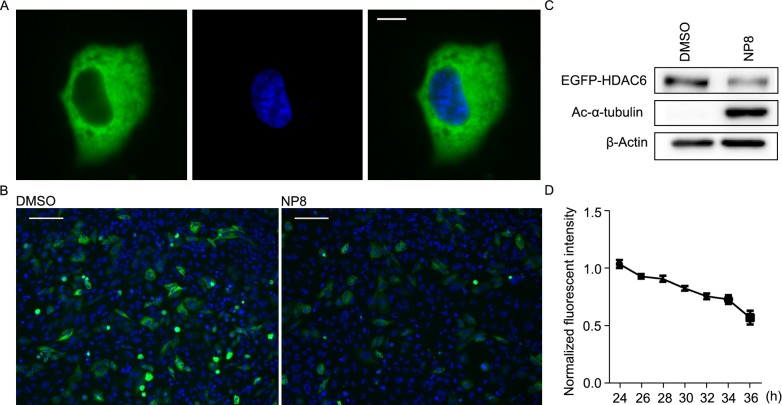
**Visualization of NP8-induced degradation**. (A) The EGFP-HDAC6 (green) was localized in the cytoplasm of HeLa cells. Scale bar: 10 µm. (B) NP8 treatment caused reduced fluorescence signal within 48 h. Nuclei were labeled by Hoechst 33342 (blue). Scale bar: 50 µm. (C) Confirmation of EGFP-HDAC6 degradation in (B) by immunoblotting. (D) Relative mean fluorescence intensity of representative single cells was monitored via confocal microscope from 24 h to 36 h post 5 µmol/L NP8 treatment

In summary, by conjugating a novel HDAC6 inhibitor Nex A with CRBN ligand Poma, we have developed a new class of PROTAC degraders of HDAC6. Among the different HDAC6-targeting PROTACs we developed, NP8 stood out as the most efficient degrader. NP8 effectively induced degradation of HDAC6 at 100 nmol/L in different cell lines, most significantly in multiple myeloma cells. NP8-induced degradation of HDAC6 was a rapid and specific process which required the simultaneous binding of HDAC6 and CRBN and was dependent on proteasome activity. The EGFP-fused HDAC6 was responsive to NP8-induced degradation, suggesting the potential combination of protein degrader with fluorescence techniques to monitor the dynamics of interested proteins. In addition, the comparable inhibitory activity of NP8 and Nex A on multiple myeloma cells implies the future application of novel HDAC6-degradation strategy to treat this notorious disease. Further visualization attempt and functional studies of NP8 *in vivo* is currently in progress in our laboratory.

## Electronic supplementary material

Below is the link to the electronic supplementary material.

Supplementary material 1 (PDF 1364 kb)
