# Correction to: Developing potent PROTACs tools for selective degradation of HDAC6 protein

**DOI:** 10.1007/s13238-019-0613-4

**Published:** 2019-02-22

**Authors:** Zixuan An, Wenxing Lv, Shang Su, Wei Wu, Yu Rao

**Affiliations:** 1 MOE Key Laboratory of Protein Sciences, School of Life Sciences, Tsinghua University, Beijing, 100084 China; 2 MOE Key Laboratory of Protein Sciences, School of Pharmaceutical Sciences, MOE Key Laboratory of Bioorganic Phosphorus Chemistry & Chemical Biology, Beijing Advanced Innovation Center for Structural Biology, Tsinghua University, Beijing, 100084 China

## Correction to: Protein Cell 10.1007/s13238-018-0602-z

In the original publication the title of X axis in Fig. 1G is incorrectly published as “Compound (µmol/L)”. The correct title of X axis in Fig. 1G should be read as “Compound (nmol/L)”

**Figure 1:**
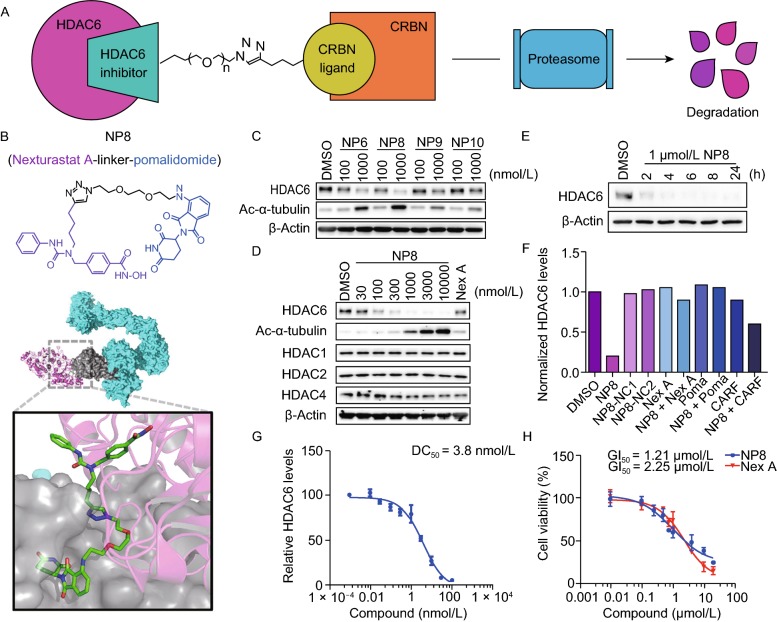
**Development of selective HDAC6-degrading PROTACs**. (A) The principle of PROTAC. (B) The structure of PROTAC, as shown in the upper portion. A binding mode of PROTAC (ball stick), HDAC6 (PDB 5G0J, purple) and CRL4-CRBN (PDB 2HYE and 4CI3, colored cyan and gray) was simulated by Pymol. (C) Screen for a potent HDAC6 degrader. HeLa cells were treated as indicated for 24 h. (D) Characterization of NP8-induced degradation in HeLa cells. The degradation of HDAC6 was in a dose-dependent manner. The HDAC1, HDAC2 and HDAC4 levels were not affected within 24 h. (E) NP8 caused fast degradation of HDAC6 within 2 h in HeLa cells. (F) NP8-NC1 and NP8-NC2 failed to degrade HDAC6. NP8 induced degradation was rescued by single introduction of Nexturastat A (Nex A, 300 nmol/L), Pomalidomide (Poma, 10 µmol/L) or Carfilzomib (CARF, 1 µmol/L). The concentration of NP8-NC1, NP8-NC2 and NP8 were 300 nmol/L. HeLa cells were treated with Carfilzomib for 6 h and 24 h for the rest. (G) The degradation of HDAC6 by titration of NP8 for 24 h in MM.1S cells. (H) MM.1S cells were treated with NP8 or Nexturastat A (Nex A) for 72 h. Cell viability was determined by CCK-8 assay (*n* = 3)

