# Supplementary material for: Developing potent PROTACs tools for selective degradation of HDAC6 protein
Source: Protein Cell. 2019 Jan 2;10(8):606–9. doi: 10.1007/s13238-018-0602-z (PMC6626596; doi:10.1007/s13238-018-0602-z)

MATERIALS AND METHODS

Cell lines and Reagents. All the cell lines applied in this article (HeLa, A549, hTERT-RPE1, U251, Jurkat, HCT116, RPMI 8226 and MM.1S) were purchased from Cell Bank and Stem Cell Bank, Chinese Academy of Sciences and maintained as instructed.

For primary antibodies, antibodies against HDAC6 (7558S), HDAC1 (5356T), HDAC2 (5113S), HDAC4 (5392T) were purchased from Cell Signaling Technology and applied in 1:1000 dilution. GFP antibody (M20004, 1:5000 dilution) were purchased from Abmart. Acetylated α-Tubulin antibody (T7451, 1:5000 dilution) was purchased from Sigma Aldrich. β-Actin antibody (60008-1-Ig, 1:5000 dilution) was purchased from Protein Tech. HDAC10 antibody (ab108934) was purchased from Abcam.

HRP-conjugated secondary antibodies goat-anti-mouse IgG and goat-anti-rabbit IgG were purchased from Thermo Scientific and applied with 1:10000 and 1:5000 dilution separately. Pomalidomide (S1567) was purchased from Selleck. Carfilzomib (A1933) was purchased from ApexBio. Nexturastat A was synthesized according to the methods published.

Immunoblotting. Cells were seeded in 24-well plates, incubated overnight and treated with com-pounds at indicated concentrations for given time. To prepare cell samples for immunoblotting, cells were collected and lysed on ice by 45 μL RIPA buffer (50 mM Tris-HCl pH7.4, 150 mM NaCl, 1% NP-40, 0.5% SDS) and then mixed with 15 μL 4X SDS loading buffer (200 mM Tris-HCl pH6.8, 400 mM DTT, 0.04% Bromophenol Blue, 8% SDS, 40% Glycerin) before heated at 95℃ for 10 min. Immunoblotting was performed by standard protocol. In brief, 6 μL protein samples were loaded into the gel and the voltages were set to 80 V for stacking gel phase and 120 V for separating gel phase. After 1.5 h, proteins were transferred from gels onto PVDF membranes in ice-water bath for another 1.5 h under constant current mode. The membranes were then blocked with 5% non-fat milk in 1X TBST at room temperature for 1 h and incubated with certain primary antibodies at 4℃ overnight. The incubated membranes were washed with 1X TBST for 10 min at room temperature three times before incubation with secondary antibodies for 1 h at room temperature. After washing for another three times, membranes were incubated with ECL reagent (29050 from Engreen) and photographed with MiniChemi system (Sage Creation). The experiments were repeated for at least three times. Band intensities were quantified by ImageJ 1.51. DC_{50} was calculated by GraphPad Prism 6.

Proliferation Assay. MM.1S cells were seeded in 96-well tissue culture plates (10000 cells/100 μL). The stock compound was added to cell suspension at indicated concentration before seeding. Four replicates were set in each group. After 72 h, 10 μL per well of CCK-8 solution (A311 from Vazyme) was added and allowed to incubate at 37 ℃ for 2 h before plate reading on SpectraMax Plus Microplate Reader (SoftMax Pro 6) at 450 nm. Absorbance or reading values were first corrected by substracting the blank medium reads and then normalized to DMSO-treated controls. The experiment was repeated three times. GI_{50} was calculated by GraphPad Prism 6.

Plasmid Construction. The human HDAC6 CDS was cloned from the pGEX-HDAC6 template and ligated into the pEGFP-C1 by restriction enzyme sites (HindIII and XbaI) to construct the expression plasmid pEGFP-HDAC6. The plasmid was confirmed by sequencing.

Visualization. The pEGFP-HDAC6 (100 ng) was transfected into HeLa cells (cultured in 24-well plates) using Vigofect regents. After 12 h, the culture medium was refreshed to new medium containing 10 μM NP8 or DMSO and cells were allowed to grow for another 48 h. Photos were taken under Olympus Inverted Fluorescence Microscope with 10x (air) and 40x (air) objective. Cells were lysed by 2X SDS loading buffer for immunoblotting analysis.

For single cell visualization, HeLa cells were transfected with the pEGFP-HDAC6 (50 ng) and cultured for 6 h. 5 μM NP8 was then introduced to treat transfected cells for the next 36 h. Time-lapse photos were taken every 1 hour under Olympus Spindisk Confocal with 40x (air) objectives from 24 h to 36 h post NP8 introduction. Mean fluorescence intensity was analyzed by ImageJ 1.51.

Chemistry. All commercial materials were used without further purification. Compounds **1a**, **1b**, **1e** and **1g** are commercial availiable (Alfa Aesar, Aladdin, J&K Chemical LTD.). All solvents were analytical grade. The amine **1j** was prepared by Weil's procedure(Ng, D. Y. et al., 2014). Pomalidomide derivatives **2** - **6** were prepared by Crew's procedure(Lu, J. et al., 2015). All polyethylene glycols were purchased. The ^{1}H NMR and ^{13}C NMR spectra were recorded on a Bruker AVANCEIII400 MHz spectrometer in $(CD_3)_2SO$. All ^{13}C NMR spectra were recorded with complete proton decoupling. NMR spectra are presented as chemical shifts in ppm relative to the solvent with multiplicities reported as s (singlet), d (doublet), dd (doublet of doublets), t (triplet), m (multiplet). Low-resolution mass spectral analyses were performed with an Agilent 6340 or Waters AQUITY UPLCTM/MS. Analytical TLC was performed on Yantai Chemical Industry Research Institute silica gel 60 F254 plates and flash column chromatography was performed on Qingdao Haiyang Chemical Co. Ltd silica gel 60 (200-300mesh). The rotavapor was BUCHI's Rotavapor R-3.

SUPPLEMENTARY INFORMATION

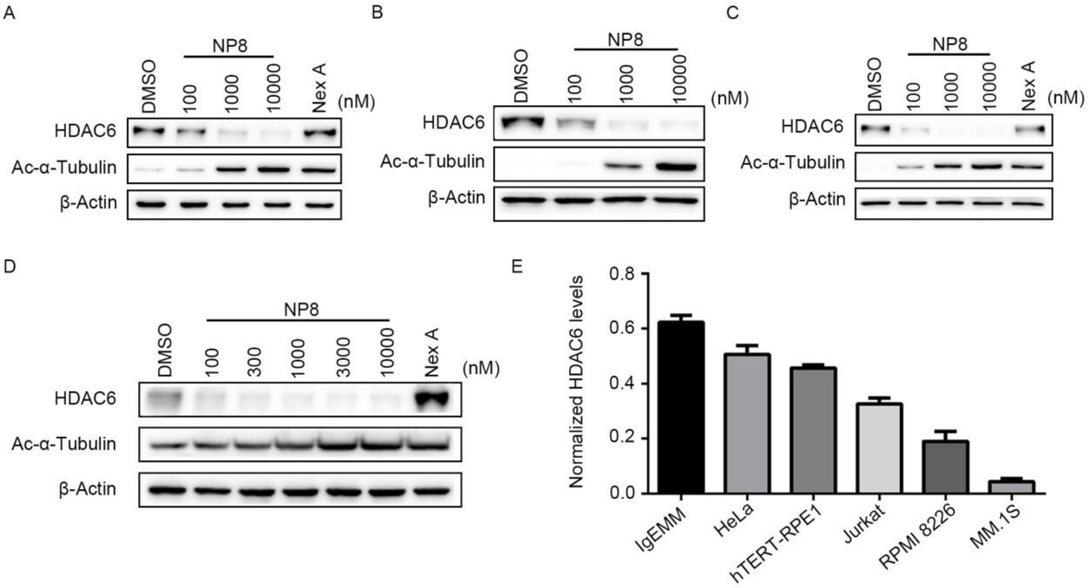

Figure S1. The effect of NP8 on intracellular HDAC6 levels of of different cell lines.

(A-D) Different human cell lines were treated with different concentrations of NP8 or 1 μM Nex A for 24 h. Then cells were harvested and lysed for immunoblotting. A, human multiple myeloma cell line IgEMM; B, human retina pigmented epithelial cell immortalized with hTERT, hTERT-RPE1; C, human multiple myeloma cell line RPMI 8226; D, human T lymphocyte cell line (leukemia) Jurkat.

(E) Relative HDAC6 levels in cells incubated with 100 nM NP8 for 24 h compared to DMSO-treated control group. Data from Figure S1 A-D and Figure 1 C and G. Band intensities were analyzed for HDAC6 levels (*n* = 3) with Image J.

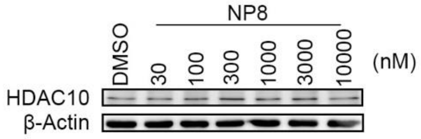

Figure S2. The effect of NP8 on the HDAC10 level in HeLa cells.

HeLa cells were incubated with NP8 as indicated for 24 h. Then cells were harvested and lysed for immunoblotting.

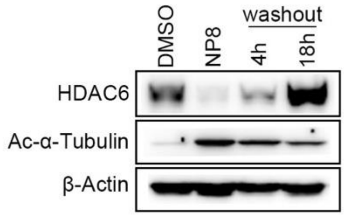

Figure S3. HDAC6 levels were restored upon NP8 washout in NP8-treated cells. HeLa cells were treated with 1 μM NP8 for 24 h, then washed three times with fresh medium to remove the drug completely (0 h) and then refreshed with medium without drug for an additional 4 h or 18 h. Total cell lysates were collected and prepared for immunoblot analysis.

Compound synthesis

Scheme 1. Synthesis of Representative PROTACs.

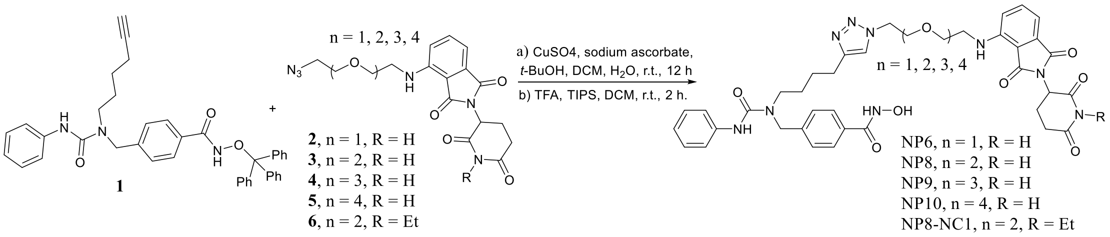

General procedure for synthesis of NP6, NP8, NP9, NP10, NP8-NC1.

A mixture of alkyne **1** (13 mg, 0.02 mmol), Pomalidomide derivatives **2** - **6** (0.02 mmol), $CuSO_4$ (3 mg, 0.02 mmol), sodium ascorbate (12 mg, 0.06 mmol), DCM (1 mL), H_2O (160 μL) in *t*-BuOH (1.6 mL) were stirred in a round bottom flask for 5 h at r.t. under Ar. The reaction was quenched by water and the mixture was washed once with saturated aqueous $NaHCO_3$, extracted by DCM. The organic layer was dried over anhydrous Na_2SO_4, and evaporated in vacuum to afford the crude product, which was further purified by silica gel column chromatography (DCM: MeOH = 20: 1) to give the 1, 4-substituted triazole derivatives respectively (IY: 40% - 50%). The 1, 4-substituted triazole intermediates (0.02 mmol) were dissolved in DCM (5 mL) in a round bottom flask respectively. Then, TIPS (0.5 mL) was added and the resulting mixture was stirred at r.t. for 10 minutes followed by added TFA (0.5 mL) and stirred for 2 h at r.t.. The reaction were quenched with $NaHCO_3$ solution and extracted with DCM. The organic layer was dried over anhydrous Na_2SO_4, and evaporated in vacuum to afford the crude product, which was further purified by silica gel column chromatography (DCM: MeOH = 10: 1) to give the **NP6**, **NP8**, **NP9**, **NP10**, **NP8-NC1** respectively. IY: 85% - 90%.

4-((1-(4-(1-(2-(2-((2-(2,6-dioxopiperidin-3-yl)-1,3-dioxoisoindolin-4yl)amino)ethoxy) ethyl)-1H-1, 2, 3-triazol-4-yl)butyl)-3-phenylureido)methyl)-N-hdroxy-benzamide (**NP6**). ^{1}H-NMR (400 MHz, DMSO-d_6) δ (ppm) 11.19 (s, 1H), 11.12 (s, 1H), 9.03 (s, 1H), 8.37 (s, 1H), 7.72-7.70 (m, 3H), 7.54 (t, J = 7.4Hz, 1H), 7.45 (d, J = 8.0Hz, 2H), 7.30 (d, J = 8.0Hz, 2H), 7.20 (t, J = 7.6Hz, 2H), 7.09 (d, J = 8.0Hz, 1H), 7.03 (d, J = 8.0Hz, 1H), 6.93 (t, J = 7.4Hz, 1H), 6.58 (t, 1H), 5.05 (dd, J = 12.8 Hz, J = 5.2 Hz, 1H), 4.59 (s, 2H), 4.49-4.42 (m, 2H), 3.81-3.78 (m, 2H), 3.58-3.56 (m, 2H), 3.50-3.49 (m, 2H), 3.44-3.38 (m, 2H), 3.40-3.31(m, 2H), 2.92-2.81 (m, 1H), 2.50-2.49 (m, 4H), 2.02-1.98 (m, 1H), 1.52 (s, 4H); LC-MS: calculated for $C_{38}H_{41}N_9O_8$ $[M+H]^+$, 752.30; found, 752.63.

4-((1-(4-(1-(2-(2-(2-((2-(2,6-dioxopiperidin-3-yl)-1,3-dioxoisoindolin-4-yl)amino)eth oxy)etoxy)ethyl)1H-1, 2, 3-triazol-4-yl)butyl)-3-phenylureido)methyl)-N-hydroxy benzamide (**NP8**). ^{1}H-NMR (400 MHz, DMSO-d_6) δ (ppm) 11.17 (s, 1H), 11.10 (s, 1H), 9.01 (s, 1H), 8.38 (s, 1H), 7.74-7.68 (m, 3H), 7.56 (t, J = 7.4Hz, 1H), 7.46 (d, J = 8.0Hz, 2H), 7.30 (d, J = 8.0Hz, 2H), 7.21 (t, J = 7.6Hz, 2H), 7.10 (d, J = 8.6Hz, 1H), 7.03 (d, J = 7.0Hz, 1H), 6.93 (t, J = 7.4Hz, 1H), 6.60-6.57 (m, 1H), 5.04 (dd, J = 12.8 Hz, J = 5.2 Hz, 1H), 4.59 (s, 2H), 4.43-4.41 (m, 2H), 3.78-3.75 (m, 2H), 3.57-3.54 (m, 2H), 3.50 (s, 4H), 3.44-3.4 (m, 2H), 3.40-3.31(m, 2H), 2.91-2.82 (m, 1H), 2.50-2.49 (m, 4H), 2.02-1.97 (m, 1H), 1.53 (s, 4H); ^{13}C-NMR (100 MHz, DMSO-d_6) δ (ppm) 172.8, 170.1, 168.9, 167.3, 155.9, 146.5, 146.4, 142.3, 140.4, 136.3, 132.1, 131.6, 131.4, 129.7, 128.7, 128.2, 127.0, 126.9. 122.1, 121.9, 120.1, 117.4, 110.7, 109.2, 69.6, 69.5, 68.9, 68.8, 49.2, 48.9, 48.6, 46.0, 41.7, 30.9, 27.3, 26.2, 24.7, 23.3, 22.4, 22.1; LC-MS: calculated for $C_{40}H_{45}N_9O_9$ $[M+H]^+$, 796.33; found, 796.67.

4-((1-(4-(1-(2-(2-(2-(2-((2-(2,6-dioxopiperidin-3-yl)-1,3-dioxoisoindolin-4-yl)amino) ethoxy)ethoxy)ethoxy)ethyl)-1H-1,2,3-triazol-4-yl)butyl)-3-phenylureido)methyl)-N-hydroxybenzamide (**NP9**). ^{1}H-NMR (400 MHz, DMSO-d_6) δ (ppm) 11.21 (s, 1H), 11.11 (s, 1H), 9.06 (s, 1H), 8.39 (s, 1H), 7.75-7.70 (m, 3H), 7.55 (t, J = 7.4Hz, 1H), 7.45 (d, J = 8.0Hz, 2H), 7.31 (d, J = 8.0Hz, 2H), 7.21 (t, J = 7.6Hz, 2H), 7.11 (d, J = 8.6Hz, 1H), 7.01 (d, J = 7.0Hz, 1H), 6.94 (t, J = 7.4Hz, 1H), 6.58-6.56 (m, 1H), 5.04 (dd, J = 12.8 Hz, J = 5.2 Hz, 1H), 4.59 (s, 2H), 4.43-4.40 (m, 2H), 3.76-3.73 (m, 2H), 3.57-3.50 (m, 12H), 3.40-3.32 (m, 2H), 2.89-2.82 (m, 1H), 2.58-2.55 (m, 4H), 2.00-1.98 (m, 1H), 1.52 (s, 4H); LC-MS: calculated for $C_{42}H_{49}N_9O_{10}$ $[M+H]^+$, 840.36; found, 840.63.

4-((1-(4-(1-(14-((2-(2, 6-dioxopiperidin-3-yl)-1, 3-dioxoisoindolin-4-yl)amino)-3, 6, 9, 12-tetraxatetradecyl)-1H-1,2,3-triazol-4-yl)butyl)-3-phenylureido)methyl)-N-hydroxy benzamide (**NP10**). ^{1}H-NMR (400 MHz, DMSO-d_6) δ (ppm) 11.20 (s, 1H), 11.12 (s, 1H), 9.05 (s, 1H), 8.39 (s, 1H), 7.76-7.70 (m, 3H), 7.56 (t, J = 7.4Hz, 1H), 7.45 (d, J = 8.0Hz, 2H), 7.31 (d, J = 8.0Hz, 2H), 7.21 (t, J = 7.6Hz, 2H), 7.11 (d, J = 8.6Hz, 1H), 7.02 (d, J = 7.0Hz, 1H), 6.93 (t, J = 7.4Hz, 1H), 6.59-6.57 (m, 1H), 5.04 (dd, J = 12.8 Hz, J = 5.2 Hz, 1H), 4.59 (s, 2H), 4.43-4.41 (m, 2H), 3.76-3.73 (m, 2H), 3.59-3.43 (m, 16H), 3.40-3.32 (m, 2H), 2.91-2.81 (m, 1H), 2.59-2.49 (m, 4H), 2.02-1.97 (m, 1H),

1.51 (s, 4H); LC-MS: calculated for $C_{44}H_{53}N_9O_{11}$ $[M+H]^+$, 884.38; found, 884.67.

4-((1-(4-(1-(2-(2-(2-((2-(1-ethyl-2,6-dioxopiperidin-3-yl)-1,3-dioxoisoindolin-4-yl)amino)ethoxy)eth-oxy)ethyl)-1H-1,2,3-triazol-4-yl)butyl)-3-phenylu-reido)methyl)-N-hydroxybenzamide (**NP8-NC1**). ^{1}H-NMR (400 MHz, DMSO-d_6) δ (ppm) 10.15 (s, 1H), 9.02 (s, 1H), 8.38 (s, 1H), 7.73-7.69 (m, 3H), 7.55 (t, J = 7.4Hz, 1H), 7.44 (d, J = 8.0Hz, 2H), 7.30 (d, J = 8.0Hz, 2H), 7.21 (t, J = 7.6Hz, 2H), 7.10 (d, J = 8.6Hz, 1H), 7.02 (d, J = 7.0Hz, 1H), 6.93 (t, J = 7.4Hz, 1H), 6.59-6.57 (m, 1H), 5.10 (dd, J = 12.8 Hz, J = 5.2 Hz, 1H), 4.57 (s, 2H), 4.43-4.40 (m, 2H), 3.76-3.73 (m, 2H), 3.59-3.43 (m, 2H), 3.50 (s, 4H) 3.44-3.41 (m, 2H), 3.40-3.31(m, 2H), 3.30-3.29 (m, 2H), 2.91-2.81 (m, 1H), 2.59-2.49 (m, 4H), 2.02-1.98 (m, 1H), 1.52 (s, 4H), 0.97(t, J = 7.0Hz, 3H); LC-MS: calculated for $C_{44}H_{53}N_9O_{11}$ $[M+H]^+$, 824.36; found, 824.68.

Scheme 2. Synthesis of Alkyne 1.

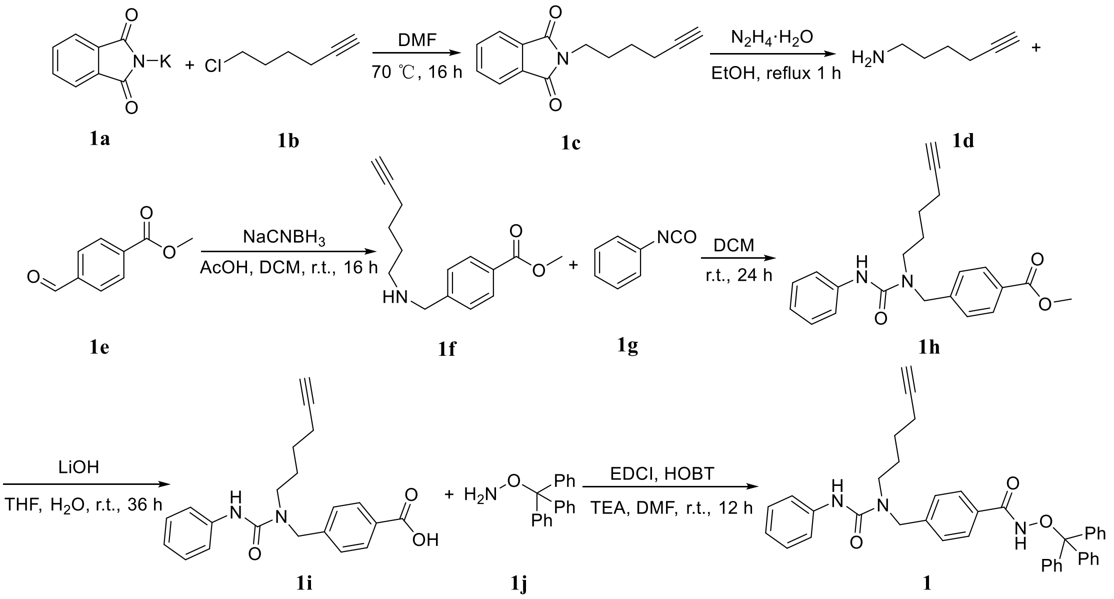

2-(hex-5-yn-1-yl)isoindoline-1, 3-dione (1c). A mixture of **1a** (1.2 g, 6.4 mmol) and **1b** (466 mg, 4 mmol) in DMF 10 mL was stirred in a round bottom flask at 70°C for 16 h. The reaction mixture was cooled to r.t. and extracted by EtOAc. The organic layer was dried over anhydrous Na_2SO_4, and evaporated in vacuum to afford the crude product, which was further purified by silica gel column chromatography (petroleum ether: ethyl acetate = 4: 1) to give the intermediate **1c**, IY: 80%.

hex-5-yn-1-amine (1d). A round bottom flask was charged with **1c** (700 mg, 3 mmol) and 50 mL EtOH. Hydrazine monohydrate (530 mg, 10 mmol) was added to the white suspension until became homogeneous. The colorless solution was heated at 70°C for 2 h, during which time a white solid precipitated. The solution was cooled to r.t. 75 mL H_2O was added to solution, followed by 2N HCl to adjust PH to ~3.5. The mixture was filtered to remove the white precipitate. The filtrate was concentrated under reduced pressure to give the intermediate **1d**.

methyl 4-((hex-5-yn-1-ylamino)methyl)benzoate (1f). A mixture of **1d** (485 mg, 5 mmol) and **1e** (821 mg, 5 mmol) in a solution of 5% AcOH in DCM (20 mL) was stirred in a round bottom flask at 0°C for 5 min. Then, sodium cyanoborohydride was added in portions and the resulting mixture was stirred at r.t. under an atmosphere of Ar overnight. The reaction was quenched with 2N NaOH. The aqueous layer was extracted with DCM. The organic layer was dried over anhydrous Na_2SO_4, and evaporated in vacuum to afford the crude product, which was further purified by silica gel column chromatography(petroleum ether: ethyl acetate = 1： 1) to give the intermediate **1f** , IY: 59%.

methyl 4-((1-(hex-5-yn-1-yl)-3-phenylureido)methyl)benzoate (1h). A solution of **1f** (508 mg, 2 mmol) in DCM was added the appropriate **1g** (247 mg, 2 mmol) at r.t. under an Ar atmosphere, and the resulting solution was stirred at r.t. overnight. The reaction was quenched with saturated bicarbonate and extracted with DCM. The combined organic layer were washed with brine, and evaporated in vacuum to afford the crude product, which was further purified by silica gel column chromatography(petroleum ether: ethyl acetate = 2: 1) to give the intermediate **1h**, IY: 85%.

4-((1-(hex-5-yn-1-yl)-3-phenylureido)methyl)benzoic acid (1i). A round bottom flask was charged with **1h** (364 mg, 1 mmol) and THF 10 mL. A solution of LiOH (115 mg, 5 mmol) in water was added. The mixture was stirred at r.t. for 36 h. 2N HCl was added to adjust PH to ~3.5. The solution was extracted with DCM and the organic layer were evaporated in vacuum to afford the intermediate **1i**, which was used for next step without further purification.

4-((1-(hex-5-yn-1-yl)-3-phenylureido)methyl)-N-(trityloxy)benzamide (1). A mixture of **1i** (350 mg, 1 mmol), **1j** (275 mg, 1 mmol), HOBT (162 mg, 1.2 mmol), EDCI (230 mg, 1.2 mmol) and Et_3N (166 μL, 1.2 mmol) in dry DMF (10 mL) was stirred in a round bottom flask for 24 h at r.t.. The reaction was quenched by water and the mixture was washed once with saturated aqueous NaCl, extracted by EtOAc. The organic layer was dried over anhydrous Na_2SO_4, and evaporated in vacuum to afford the crude product, which was further purified by silica gel column chromatography (petroleum ether: ethyl acetate = 1: 1) to give the **1**, IY: 71%.

Scheme 3. Synthesis of Pomalidomide derivatives 2 – 6.

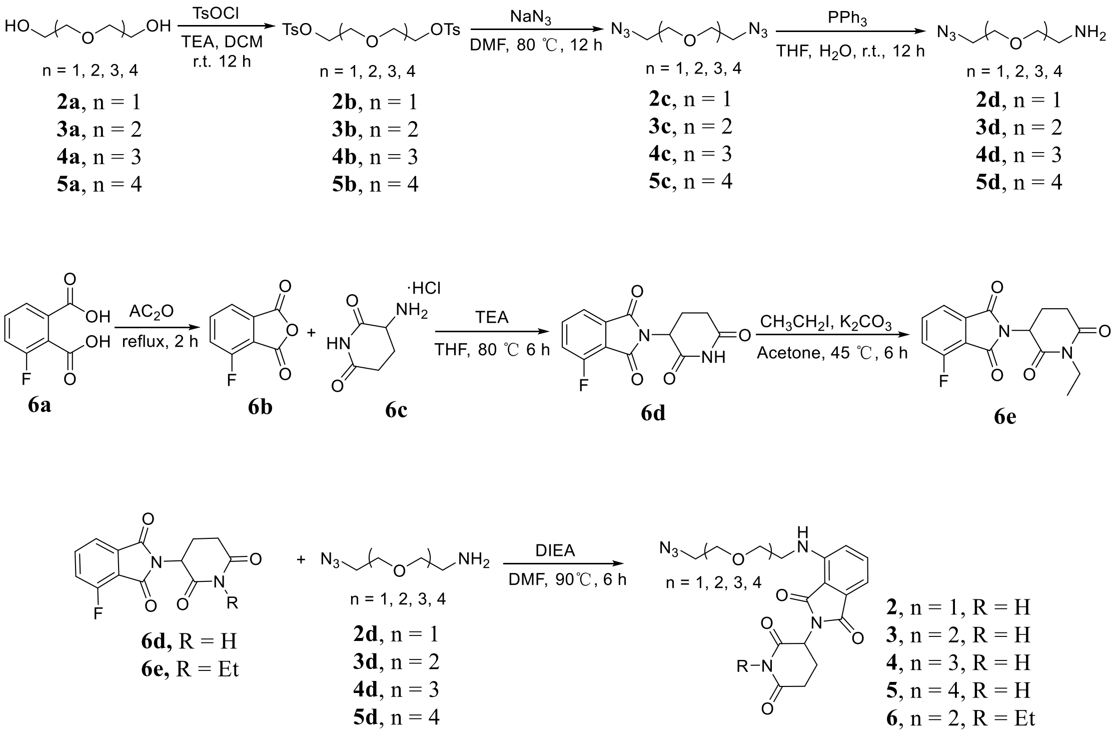

General procedure for synthesis of 2d - 5d.

A round bottom flask was charged with diethylene glycol **2a** (4.5 g, 40 mmol) , TEA (14 mL) and DCM (50 mL). 4-toluene sulfonyl chloride (17 g, 88 mmol) was added in portion. The mixture was stirred at r.t. for 12 h. The reaction was quenched with 2N NaOH. The aqueous layer was extracted with DCM. The organic layer was dried over anhydrous Na_2SO_4, and evaporated in vacuum to afford the crude product, which was further purified by silica gel column chromatography (petroleum ether: ethyl acetate = 4： 1) to give the intermediate **2b** , IY: 85%. Then **2b** (12.5 g, 30 mmol) was dissolved in DMF (50 mL) and added sodium azide (7.8 g, 120 mmol) in portion. The resulting mixture was heated to 80°C and kept stirring for another 12 h. The reaction mixture was cooled to r.t. and extracted by EtOAc. The organic layer was dried over anhydrous Na_2SO_4, and evaporated in vacuum to afford the crude product, which was further purified by silica gel column chromatography (petroleum ether: ethyl acetate = 4: 1) to give the intermediate **2c**, IY: 92%. Next, **2c** (4.6 g, 30 mmol) was dissolved in THF (40 mL) and H_2O (10 mL) at r.t.. Triphenylphosphine (8.5 g, 30 mmol) was added in portion. The resulting mixture was kept stirring for 12 h. Then the reaction was quenched with 2N HCl and extracted with DCM. The aqueous layer were collected and 2N NaOH was added to adjust PH to ~10. The solution was extracted with DCM and the organic layer were evaporated in vacuum. The crude product was further purified by silica gel column chromatography (DCM: MeOH = 30: 1) to give the **2d**, IY: 48%. The same procedure was used for synthesis **3d**, **4d** and **5d**.

2-(1-ethyl-2,6-dioxopiperidin-3-yl)-4-fluoroisoindoline-1,3-dione (6e). **6a** (2 g, 11 mmol) was dissolved in acetic anhydride (10 mL). The resulting mixture was refluxed for 2 h. The solvent was removed by vacuum. The residues were purified by silica gel column chromatography (petroleum ether: ethyl acetate = 4: 1) to give **6b**, IY: 92 %. Then **6b** (996 mg, 6 mmol) and **6c** (984 mg, 6 mmol) were dissolved in THF (20 mL). TEA (1 mL) was added. The resulting mixture was refluxed for 6 h. The solvent was removed. The residues were purified by silica gel column chromatography (petroleum ether: ethyl acetate = 1: 1) to give **6d**, IY: 69 %. Next, **6d** (277 mg, 1 mmol), iodoethane (155 mg, 1 mmol) and K_2CO_3 (140 mg, 1.1 mmol) were dissolved in acetone (10 mL). The resulting mixture were refluxed for 3 h. The reaction was allowed to cool to r.t. and quenched with saturated aqueous NH_4Cl solution and extracted with DCM, washed with water. The organic layer was dried over anhydrous Na_2SO_4, and evaporated in vacuum to afford the crude product, which was further purified by silica gel column chromatography (petroleum ether: ethyl acetate = 1: 1) to give the **6e**, IY: 65%.

General procedure for synthesis of 2 - 6.

A mixture of **6d** (277 mg, 1 mmol), **2d** (131 mg, 1 mmol), DIEA (356 μL, 2 mmol) in dry DMF (5 mL) was stirred in a round bottom flask for 6 h at 90 ^{o}C. The reaction was quenched by water and the mixture was washed once with saturated aqueous NaCl, extracted by EtOAc. The organic layer was dried over anhydrous Na_2SO_4, and evaporated in vacuum to afford the crude product, which was further purified by silica gel column chromatography (DCM: MeOH = 40: 1) to give one of the Pomalidomide derivative **2**, IY: 25%. The same procedures were used for synthesis of **3**, **4**, **5** and **6**.

References

Ng, D. Y., Arzt, M., Wu, Y., Kuan, S. L., Lamla, M., Weil, T. (2014). Constructing hybrid protein zymogens through protective dendritic assembly. Angew Chem Int Ed Engl. 53, 324-328.

Lu, J., Qian, Y., Altieri, M., Dong, H., Wang, J., Raina, K., Hines, J., Winkler, J. D., Crew, A. P., Coleman, K., Crews, C. M. (2015). Hijacking the E3 Ubiquitin Ligase Cereblon to Efficiently Target BRD4. Chem Biol. 22, 755-763.

NMR Spectrum of NP6, NP8, NP9, NP10

^{1}H-NMR of **NP8**

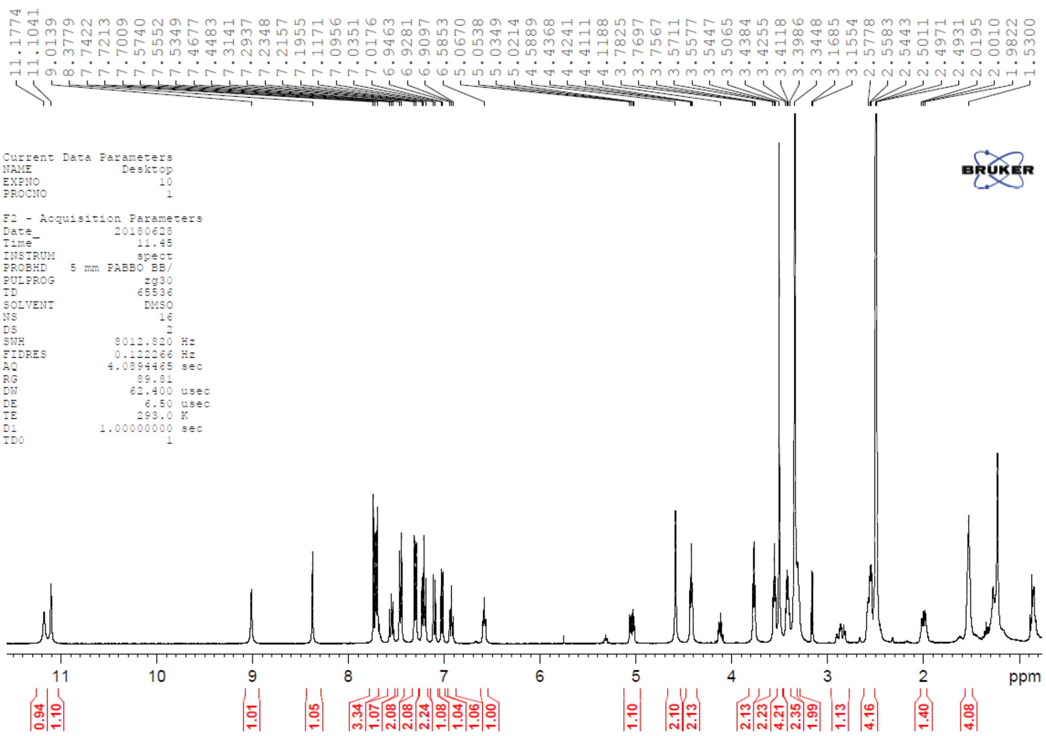

^{13}C-NMR of **NP8**

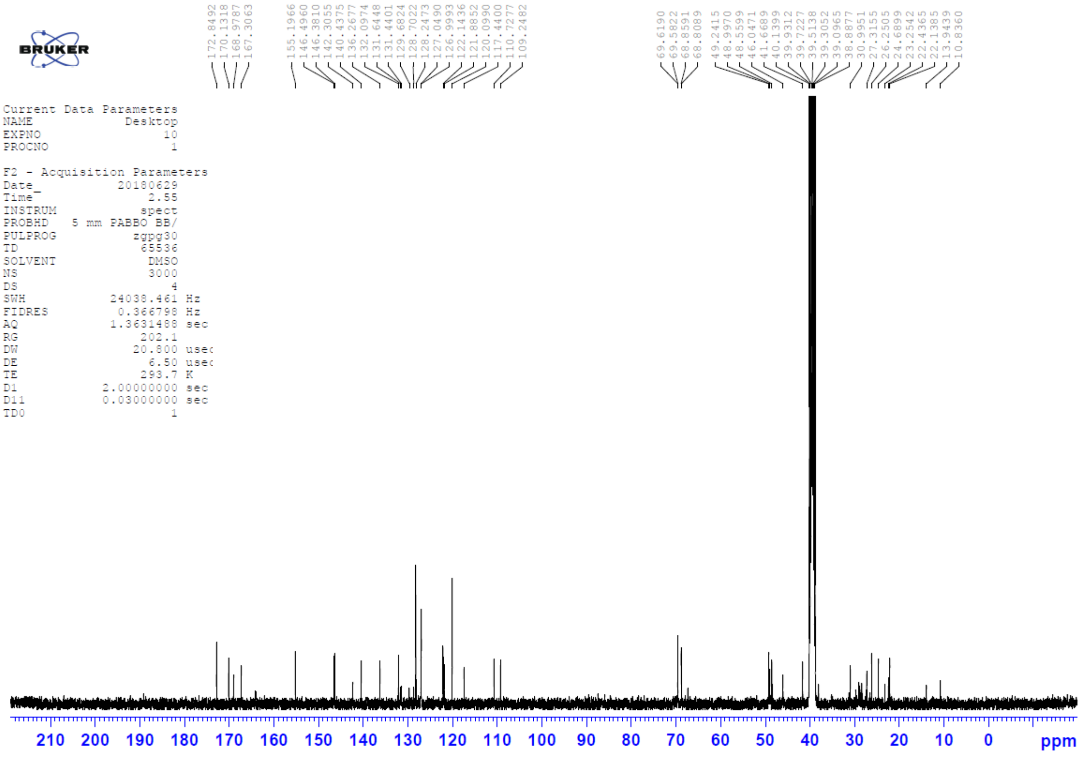

^{1}H-NMR of **NP6**

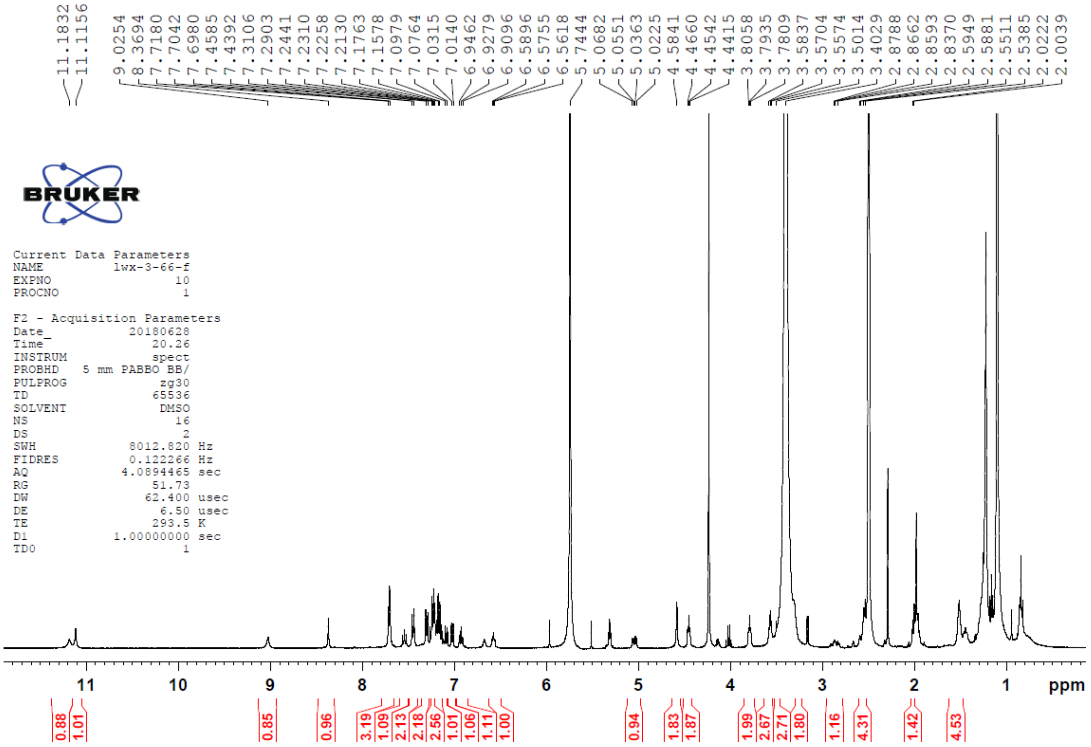

^{1}H-NMR of **NP9**

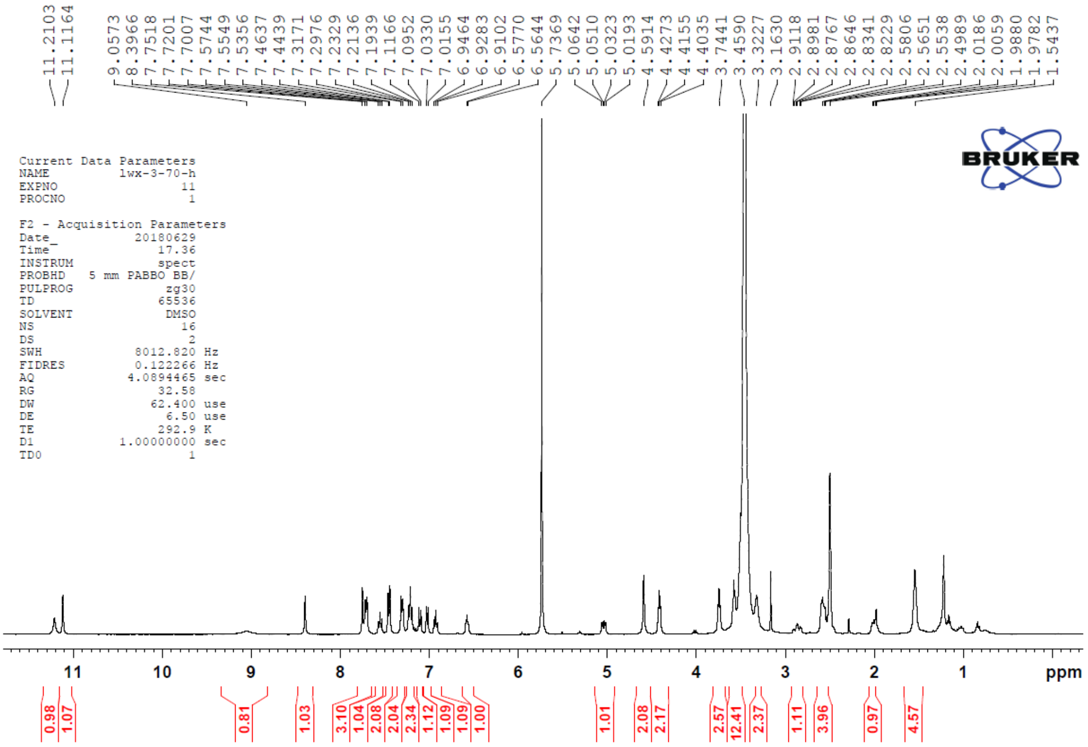

^{1}H-NMR of **NP10**

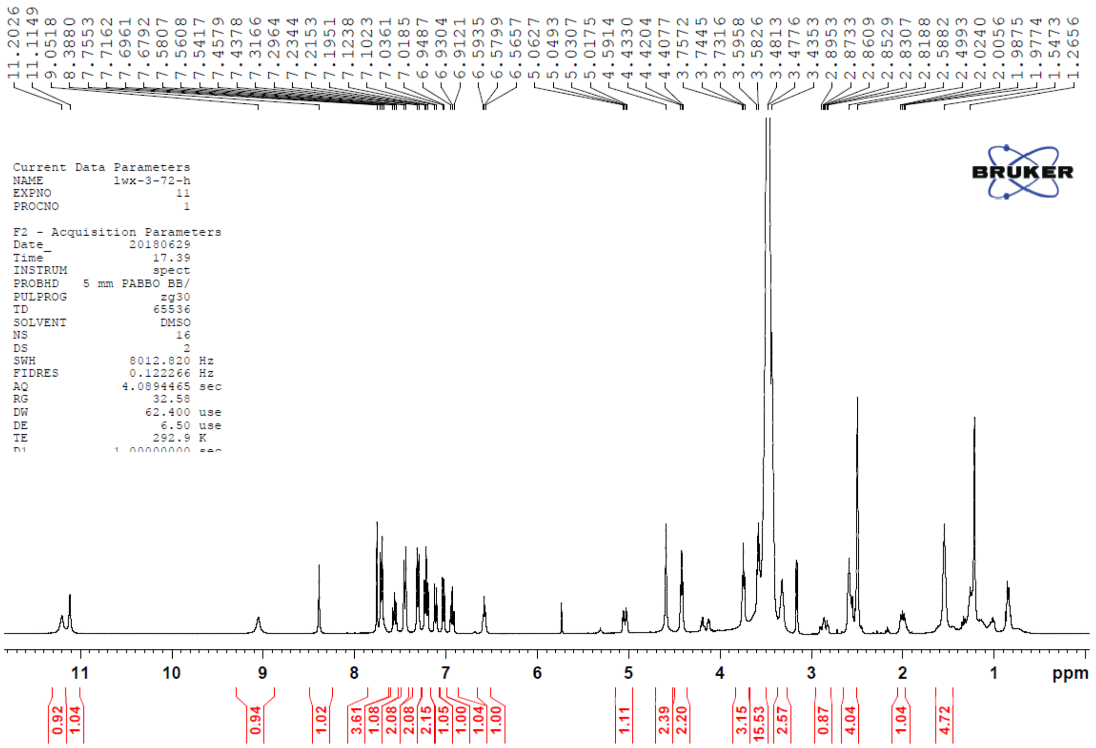

Supplement: Supplementary file 1 — Supplementary material 1 (PDF 1364 kb) [file 13238_2018_602_MOESM1_ESM.pdf]
